# Intracellular trafficking as a determinant of AS-DACA cytotoxicity in rhabdomyosarcoma cells

**DOI:** 10.1186/1471-2121-12-36

**Published:** 2011-08-24

**Authors:** Steven J Wolf, Tony Huynh, Nicole S Bryce, Trevor W Hambley, Laurence PG Wakelin, Bernard W Stewart, Daniel R Catchpoole

**Affiliations:** 1 Biospecimens Research and Tumour Bank, Children's Cancer Research Unit, The Children's Hospital at Westmead, Westmead, NSW 2774, Australia; 2 School of Chemistry, University of Sydney, Sydney, NSW 2006, Australia; 3 School of Medical Sciences, University of New South Wales, Sydney, NSW 2052, Australia; 4 Cancer Control Program, South East Sydney & Illawarra Public Health Unit, Randwick, NSW 2031, Australia

## Abstract

**Background:**

Rhabdomyosarcoma (RMS) is a malignant soft tissue sarcoma derived from skeletal muscle precursor cells, which accounts for 5-8% of all childhood malignancies. Disseminated RMS represents a major clinical obstacle, and the need for better treatment strategies for the clinically aggressive alveolar RMS subtype is particularly apparent. Previously, we have shown that the acridine-4-carboxamide derivative AS-DACA, a known topoisomerase II poison, is potently cytotoxic in the alveolar RMS cell line RH30, but is 190-fold less active in the embryonal RMS cell line RD. Here, we investigate the basis for this selectivity, and demonstrate in these RMS lines, and in an AS-DACA- resistant subclone of RH30, that AS-DACA-induced cytotoxicity correlates with the induction of DNA double strand breaks.

**Results:**

We show that inhibition of the multidrug-resistance associated protein (MRP1) has no effect on AS-DACA sensitivity. By exploiting the pH-dependent fluorescence properties of AS-DACA, we have characterized its intracellular distribution, and show that it concentrates in the cell nucleus, as well as in acidic vesicles of the membrane trafficking system. We show that fluorescence microscopy can be used to determine the localization of AS-DACA to the nuclear and cytoplasmic compartments of RMS cells grown as spheroids, penetrance being much greater in RH30 than RD spheroids, and that the vesicular signal leads the way into the spheroid mass. EEA1 and Rab5 proteins, molecular markers expressed on early-endosomal vesicles, are reduced by > 50% in the sensitive cell lines.

**Conclusion:**

Taking the evidence as a whole, suggests that endosomal vesicle trafficking influences the toxicity of AS-DACA in RMS cells.

## Background

Rhabdomyosarcoma (RMS) is a malignant tumour derived from primitive rhabdomyeloblasts with differentiation towards skeletal muscle [1]. RMS makes up more than half of the soft tissue sarcomas in children, and accounts for 5-8% of all childhood malignancies [2]. Combination chemotherapy, following surgery, involving drugs which inhibit mitotic spindle function, poison topoisomerase II, inhibit transcription, and alkylate DNA, has resulted in five-year survival rates of about 65% for patients with localised RMS tumours [3]. However, the presence of metastatic or disseminated disease, or a diagnosis of the alveolar RMS subtype, confers a worse prognosis [4-7], with tumours often refractory to established chemotherapy. Clearly, new agents are a priority, especially for the 30-40% of paediatric patients with these malignancies who do not achieve full remission, or who relapse following therapy.

**Figure 1:**
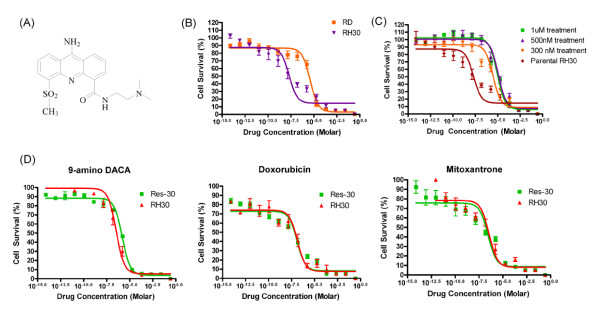
**AS-DACA resistance in rhabdomyosarcoma cells**. **(A) **Structure of AS- DACA. **(B) **MTT cell viability results presented as cytotoxicity curves for AS-DACA in RD and RH30. **(C) **Derivation of the AS-DACA-resistant RMS cell line, Res30. Dose- response curves from MTT cell viability assays performed at various stages in the development of AS-DACA-resistant RH30 cells. **(D) **Cytotoxicity curves of (i) 9-amino- DACA, (ii) doxorubicin, and (iii) mitoxantrone in Res30 cells. Error bars are SEM for independent experiments performed in triplicate.

Recently, we have shown that the acridine-4-carboxamide derivative AS-DACA (Figure 1A), elicits a marked differential response in several commonly studied rhabdomyosarcoma cell lines [8]. AS-DACA is potently cytotoxic in the RMS-derived cell line RH30, but is 190-fold less active in the RD cell line [8]. In this work we begin an investigation into the basis for this preferential activity between these two cell lines. AS- DACA is the 9-amino-5-methylsulphone derivative of the clinical candidate DACA [9,10], it has good solid tumour activity in animal models [10], intercalates into DNA as a lipohilic monocation at physiological pH with its charged N,N-dimethylethylamino side chain binding to guanine [10-12], and poisons topoisomerase II [13]. It has the unusual property amongst the acridine-4-carboxamide cytotoxins that its acridine chromophore is weakly basic with a pK of 5.2 [10], so that at pH 7.4 it is a monocation, but as the pH is lowered towards 6 and below, it becomes doubly charged. This has consequences for its fluorescence properties, which become pH-dependent, and may affect its intracellular distribution and pharmacology.

Here we use the fluorescence properties of AS-DACA to permit direct visualization of its intracellular distribution. A novel situation is therefore created whereby the cytotoxic agent itself reveals the pH of the organelle in which it is localized. Our results show that AS-DACA not only accumulates in the nucleus, as expected, but also in acidic intracellular vesicles in RMS cells. However, we find that the membrane trafficking system differs between the two RMS subtypes, with the early endosome markers, Rab5 and EEA1, expressed to a lesser degree in the sensitive RH30 cell line compared to the resistant RD cell line. Generation of an AS-DACA-resistant RH30 subline resulted in an increase in expression of the early endosome markers, Rab5 and EEA1, suggesting that the membrane trafficking system plays a significant role in the determination of AS- DACA sensitivity. In addition, we show that cytotoxic sensitivity to AS-DACA correlates with the induction of DNA double strand breaks, but is un-perturbed by the inhibition of the multidrug-resistance associated protein MRP1. Lastly, we demonstrate that fluorescence microscopy can be used to examine the intracellular distribution of AS- DACA into both the nuclear and cytoplasmic compartments of RMS cells grown as spheroids, where penetrance is much greater in RH30 than RD spheroids, and that the vesicular signal leads the way into the spheroid mass.

## Results

**Figure 2:**
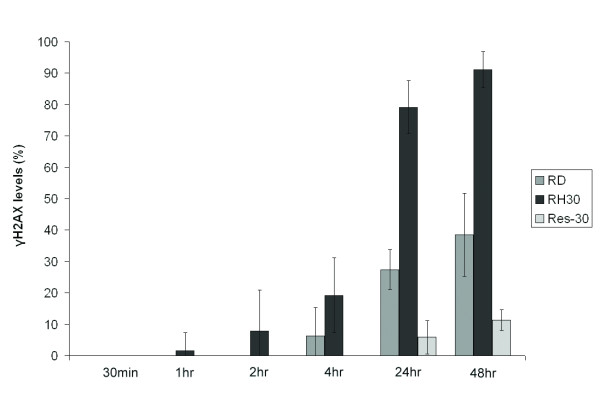
**Levels of phosphorylated H2A.X (γH2A.X) were determined using ELISA for the RMS cell lines RD, RH30, and Res30 following exposure to 20 nM of AS-DACA over time**. Levels were calculated as percentages of γH2AX detected after 48 h-exposure to 4 μM AS-DACA. Error bars are SEM for two experiments performed in triplicate providing six independent readings per value.

In previous work we showed that the IC_50 _for AS-DACA in RD cells is 3800 ± 130 nM whereas in RH30 cells it is 20 ± 0.73 nM, indicating a 190-fold difference in sensitivity (Figure 1B) [8]. To help understand the reasons for the sensitivity difference between these ERMS and the ARMS cell lines, we developed an AS-DACA-resistant sub-line of RH30 cells. Results from the MTT viability assay highlight the significant level of resistance achieved in the RH30 sub-line (Figure 1C). This cell line, designated Resistant- RH30 or "Res30", expresses a stable phenotype in which the IC_50 _for AS-DACA is 9000 nM, making it 450-fold more resistant to AS-DACA than its parent (p value = 0.009, Students t-test). Importantly, the cells retained their resistant phenotype over time. Cytotoxicity measurements reveal that Res30 cells have no cross-resistance to the topoisomerase II poisons doxorubicin and mitoxantrone (Figure 1D) (p value = 0.918 and 0.325 respectively, Students t-test), and that 9-amino-DACA, a potent cytotoxic AS- DACA analogue lacking the 5-methylsulphone group [14], shows only a limited 4.4-fold loss of potency (p value = 0.001, Students t-test). Given that AS-DACA induces cell death as a result of the accumulation of DNA double strand breaks due to the poisoning of topoisomerases [14], we investigated the level of such strand breaks induced by AS- DACA in the RMS cells by measuring the level of phosphorylation of histone H2A.X (Ser139), known as γH2A.X, which is a well-established marker of DNA double strand breaks [15,16]. Phosphorylation was assessed over 48 h in all cell lines following exposure to AS-DACA at a low dose, equivalent to the IC_50 _for RH30 cells (20nM). The results are expressed as a percentage of the DNA damage observed, compared, over the equivalent time, to that caused by a dose of 4 μM AS-DACA (Figure 2): a dose that produces maximal damage in the least sensitive RD cells. Whilst DNA damage is evident after just 1 h in the sensitive RH30 cells, its appearance is delayed 24 h in the resistant Res30 cells, and then reaches only 10% of the damage induced with a 4 μM dose of AS- DACA after 48 h: a value only ~15% of that of the parental RH30 cell line (Figure 2). Interestingly, DNA breaks are detected in RD cells after just 4 h, despite the drug concentration being approximately 190 times lower than its IC_50 _in this cell line. At 48 h the level of DNA damage reached 40% of the maximum caused by a 4 μM dose (p value = 1.25 × 10^-5^, Students t-test). These findings demonstrate that the level of DNA damage is directly proportional to the IC_50 _of AS-DACA in each cell line.

**Figure 3:**
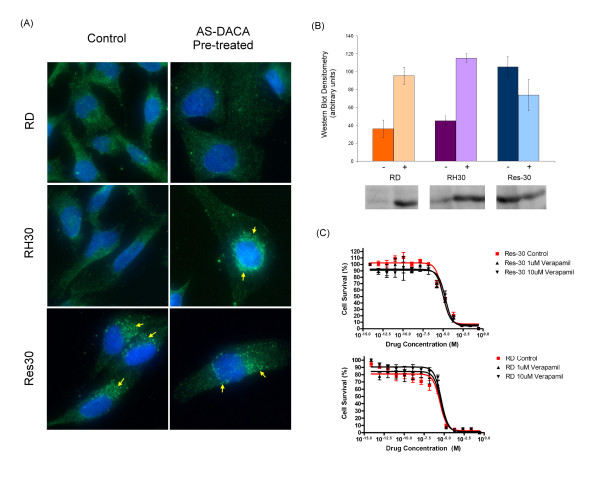
**Assessment of Multidrug Resistance Associated Protein (MRP1) in RMS cell lines**. **(A) **MRP1 expression determined by indirect immunofluorescense microscopy showing intracellular distribution of MRP1 (green) in RMS cells following treatment with AS-DACA. Co-stained with the nuclear stain DAPI (blue). **(B) **Levels of MRP1 were determined by Western blot before (-), and after (+), exposure to AS-DACA (2 × IC_50 _for 16 h). Densitometry was performed on triplicate blots with band intensity normalized for both background noise and total protein loading differences as determined by Ponceau S staining. **(C) **Influence of (R)-verapamil (1 and 10 μM) on AS-DACA cytotoxicity. RD (top) and Res30 (bottom) cells were treated for 72 h with (R)-verapamil whilst being exposed to serially increasing concentrations of AS-DACA. Error bars in (A) and (C) represent the SEM for three independent experiments.

Since AS-DACA is a known substrate of the ABC transport proteins [17], we explored the influence of a representative transporter, MRP1 (ABCC1) on AS-DACA activity in our sensitive and resistant RMS cell lines. In non-drug-treated cells, immunofluorescence microscopy of the MRP1 protein shows diffuse cytoplasmic localization in both RD and RH30 cells (Figure 3A), whereas in Res30 cells, MRP1 is localized in a punctuate fashion in the perinuclear region, suggestive of vesicular localization (arrows, Figure 3A). After 16 h exposure to AS-DACA there was no change in MRP1 localization in RD or Res30 cells, but, in contrast, MRP1 in RH30 cells now localises in a punctuate perinuclear pattern, similar to that observed in untreated Res30 cells (arrow, Figure 3A). Western blot analysis of MRP1 expression levels shows a significant rise in RD and RH30 cells following a 16 h exposure to twice the IC_50 _of AS-DACA for each cell line (8 μM and 40nM respectively) (p value = 0.02 and 0.002 respectively, Students t-test). (Figure 3B).

In contrast, although MRP1 is well-expressed in Res30 cells, its level decreases following exposure to the drug (Figure 3B). Exposure to the MRP1 inhibitor R-verapamil at concentrations of 1 and 10 μM fails to alter AS-DACA-induced cytotoxicity (Figure 3C), indicating the differential toxicity of AS-DACA in the RMS cells is not simply explained by a classical understanding of drug resistance involving MRP1.

**Figure 4:**
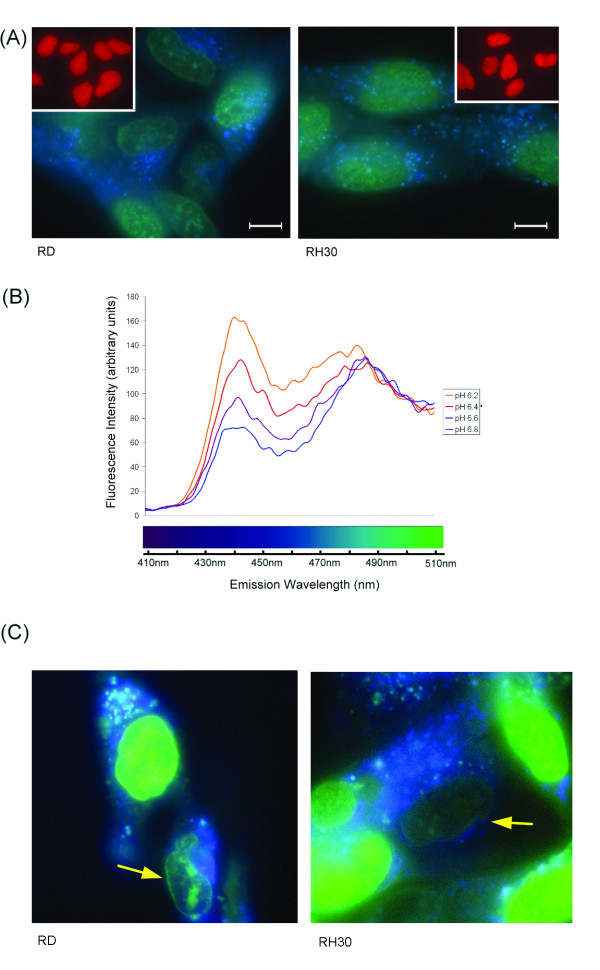
**AS-DACA fluorescence properties**. **(A) **Images of the intracellular distribution of AS-DACA in RD and RH30 cells captured using digital colour photography after a 4 h exposure to 2 μM drug. Images were captured at 600 × magnification. Scale bars represent 10 μm. Inset shows 4 h exposure to 2 μM doxorubicin. **(B) **Uncorrected fluorescence emission spectrum of AS-DACA in solution over the pH range 6.2 to 6.8. Excitation wavelength was 260 nm, λ_max _for absorption in the UV. **(C) **Fluorescent micrographs prepared using a deconvolution microscope for detection of AS-DACA. Cells were incubated for 10 min at 2 μM and show vesicle accumulation in RD (left), and RH30 (right) cells. Nuclei with reduced, or absent, staining are indicated (arrow).

Taking advantage of the fluorescent properties of AS-DACA [17] to assess its intracellular distribution in RMS cells, we exposed cells to 2 μM AS-DACA for 4 h and excited in the UV range (330-385 nm), to show, as anticipated, that the drug accumulates in the nucleus (Figure 4). Unexpectedly, however, AS-DACA was also observed to accumulate in punctate structures within the cytoplasm. As highlighted in the true colour photographs of Figure 4A, AS-DACA fluoresces green when localized to the nucleus, and blue when accumulated in small vesicle-like structures distributed throughout the cytoplasm in both cell lines. Interestingly, there is no evidence of vesicle accumulation of doxorubicin in RD and RH30 cells (Figure 4A inset), which hints at a different mechanism for accumulation, perhaps reflecting the different charge states of AS-DACA and the anthracycline. These findings prompted us to examine the fluorescence properties of AS-DACA as a function of pH, and Figure 4B shows its emission spectrum in the range 410 to 510 nm when excited in the UV at 260 nm. As the pH is lowered from 6.8 to 6.2, the green emission peak around 485 nm remains essentially unchanged, but the intensity of the blue emission at 440 nm increases substantially to become the dominant feature in this region of the spectrum. This spectral change occurs in the pH range where the acridine chromophore becomes protonated (pK of 5.2) [10], and, in consequence, provides a measure of the charged state of the ligand. Thus, with excitation in the UV, fluorescence microscopy reveals the presence of AS-DACA in the nucleus where it is bound to DNA as a monocation [12] and fluoresces green; and in acidic cytoplasmic vesicles where it is a blue-fluorescing dication (Figure 4A and 4B).

**Figure 5:**
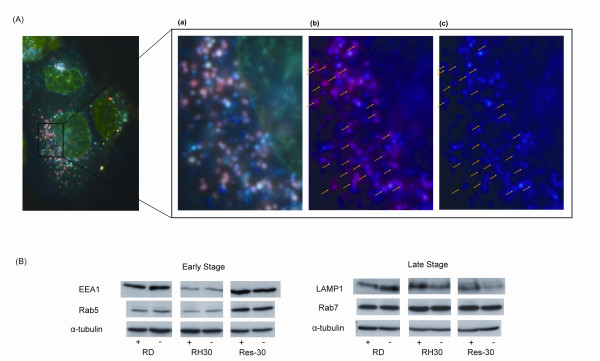
**(A) Arrows indicate co-localization of AS-DACA and LysoTracker™ Red observed in the RD cells**. (a) Digitally magnified photograph with un-altered colour. (b) The green emission has been digitally removed highlighting the pink/purple colour that results from co-localization of AS-DACA and LysoTracker™ Red. (c) The red wavelengths are then additionally removed, further highlighting sites of co-localization. **(B) **Expression levels of Rab5, Early Endosome Antigen 1 (EEA1) Rab 7 and Lysosome- associated Membrane Protein 1 (LAMP1) were determined by Western blot before (-), and after (+), exposure to AS-DACA (2 × IC_50_) for 16 h. Tubulin was included as a loading control. Shown is a single representative blot for at least three independent experiments where the same result was found in each.

We wished to determine whether the differences seen in the distribution of AS-DACA between the nucleus and vesicular structures in the two RMS cells lines is responsible for their differential sensitivity to AS-DACA toxicity. In addition, we were interested in discovering which of the cellular compartments is first to accumulate AS-DACA. Accordingly, we used a deconvolution microscope, equipped with computerized exposure control during imaging, for a more precise rendering of AS-DACA fluorescence. This was especially useful following the short treatment times designed to highlight the agents early accumulation throughout the cell. Figure 4C shows the intracellular distribution of 2 μM AS-DACA following a 10 min incubation, where it can be seen that AS-DACA can indeed be detected in both the nucleus (green) and vesicles (blue) of both RMS cell lines under these rapid-uptake conditions. The appearance of blue cytoplasmic structures occurs in both sensitive and resistant cell lines, indicative of accumulation into acidic vesicles. On rare occasions, in both cell lines, only blue vesicles can be detected, indicating vesicle accumulation prior to nuclear binding (Figure 4C arrows). To provide independent evidence that the vesicles into which AS-DACA accumulates really are acidic, we treated cells with both AS-DACA and LysoTracker™ Red, a fluorescent dye known to accumulate within acidic compartments. Co-localization of LysoTracker™ Red and AS-DACA was observed in the majority of vesicles (Figure 5A) in both cell types. However, further digital deconvolution of the microscopic images indicated that co-localization of the two agents is not always complete, with some vesicles showing only AS-DACA (blue) accumulation (Figure 5A).

The expression levels of markers specific for vesicles active at various stages of the membrane trafficking process were examined in all RMS cells, after exposure to double their IC_50 _for AS-DACA for 16 h. The expression levels of Early Endosome Antigen 1 (EEA1), Rab5, Lysosomal-Associated Membrane Protein 1 (LAMP1), and Rab7, were assessed by Western blot analysis (Figure 5B). LAMP1, which is associated with both lysosomes and late stage endosomes, and Rab7, a protein associated with late stage endosomes, were shown not to be differentially expressed in any of the cell lines, and were minimally affected by drug treatment. However, expression of EEA1, a marker of early endosomes, is substantially higher in the resistant RD and Res30 lines, and the clearly greater signal observed in Res30 cells, relative to RH30, is unperturbed by an additional overnight treatment of drug (Figure 5B). Rab5, which is also associated with early endosomes, shows similarly marked lower expression in RH30 cells relative to Res30, although, now, its expression is only marginally less than in RD cells (Figure 5B).

We hypothesized that the decrease in the amount of early endosomes in RH30 cells contributes to their sensitivity to AS-DACA, due to a lack of vesicular sequestration, which would allow the drug to pass freely throughout the cell and into the nucleus and surrounding cells. To test this, we grew RD and RH30 cells as 3-dimensional spheroids, and assessed the ability of AS-DACA to pass through multiple cell layers. Unfortunately Res30 cells were unable to be grown as spheroids. After 4 h of incubation at 20 μM, the distribution of fluorescence at both emission wavelengths across the spheroids is equivalent for both cell lines, with the intensity of nuclear green fluorescence within 10 μm of the spheroid edge being 10% greater than that of vesicular blue fluorescence. After 24 h, however, the distribution of fluorescence across the spheroids is considerably altered. The blue fluorescence is now more intense than green at the outer edge (10 μm), the difference being the most pronounced in the RD cells where the peak is ~10% below that of the RH30 cells, indicating a larger AS-DACA signal in the vesicles. Furthermore, after 24 h, AS-DACA is seen to penetrate further into the RH30 spheroids, with equivalent levels of both green and blue fluorescence being found approximately twice as deep into the cell mass as observed at 4 h. By contrast, in RD cells, both fluorescence signals are below 30% relative fluorescent intensity beyond a penetration distance of 20 μm, indicating that AS-DACA is unable to penetrate far into the RD cell mass even after a lengthy incubation. Evidently, AS-DACA traverses packed RH30 cells much more readily than packed RD cells, and it is notable that the blue vesicular signal leads the way into the spheroid masses.

## Discussion

The sensitivity of RMS cell lines to new agents may help identify novel strategies in treatment for this often refractory sarcoma. Cytotoxins based on acridine-4-carboxamide chromophores, such as the methyl sulphone derivative of N-(2-(dimethylamino)- ethyl)acridine-4-carboxamide, known as AS-DACA (Figure 1A), trap topoisomerase cleavable complexes [18,19], showing topoisomerase II activity [20]. The 190-fold differential cytotoxic response to AS-DACA detected between two RMS cell lines (Figure 1B) correlates with the delayed production of double strand breaks (Figure 2]. The immediate interpretation of this result is that AS-DACA is being prevented from intercalating with the DNA and forming cleavable complexes. With RMS being one of the most common paediatric sarcomas associated with poor drug response where the presence of metastatic or disseminated disease confers a worse prognosis [4-7], the drug resistance mechanism identified here deserves further attention. The derivation of an AS-DACA resistant RH30 cell line (Res-30) (Figure 1C) which was not cross resistant to known topoisomerase poisons (Figure 1D) suggests a resistance mechanism peculiar to AS- DACA was invoked and is suitable for comparison to the resistant RD cell line. The absence of double strand breaks in the Res-30 line (Figure 2) indicates extensive impedance of the agent from its primary cytotoxic target.

Active mechanisms that drive sequestration of weakly basic compounds into vesicles of the membrane trafficking system are not uncommon. Several of the ABC transport pumps have been implicated in such mechanisms [21,22] and alternative pathways involving the trans-golgi network [23], lung-resistance-related protein 1 (LRP1) and major vault proteins [24] have been proposed to modify intracellular drug distributions by altering transport between the nucleus, endosomal vesicles, lysosomes and the cytoplasm [25]. In 2003, Rajagopal et al [20] using HeLa cells, showed that MRP1, P-gp and BCRP were not only localized to membranes of intracellular vesicles but their roles at these sites contributed to drug resistance phenotypes via sequestration-based mechanisms. As illustrated in Figure 3 higher levels of the ABC transport protein MRP1 following treatment with AS-DACA was detected, typical of a "classical' multidrug resistance phenotype in RMS cells. However, this treatment-induced increased expression of MRP1 in cell lines does not explain the differential in sensitivity to AS-DACA induced cell death. Firstly, both RD and RH30 cell lines demonstrated an equivalent increase in MRP1 expression (Figure 3B). Interestingly, MRP1 protein was shown to localize to vesicular membranes in all three cell lines (Figure 3A) this being most prominent in cells directly after exposure to AS-DACA as well as in the derived AS-DACA resistant line Res30 pretreatment. Despite this, the absence of any effects of the MRP1 inhibitor verapamil on both the AS-DACA resistant lines (Figure 3C), indicating that impedance of MRP1 activity did not alter AS-DACA toxicity in any RMS cells.

Classically, vesicle sequestration of the anthracycline antibiotics appears to be dictated by the "partitioning theory". The partitioning theory of drug sequestration, which was proposed not long after the discovery of the lysosome in the 1960's, [25] is still believed to contribute significantly to drug resistance [26]. This theory is based upon the principle that weak acids and bases are capable of readily diffusing lipid bilayer membranes down pH gradients, rendering these compounds able to accumulate in acidic vesicles due to protonation by free H+ ions present in the vesicular lumen. Such ionization renders the drug impermeable to the membrane and accumulation will continue whilst vesicular H+ ion concentrations are maintained [23,25-27].

**Figure 6:**
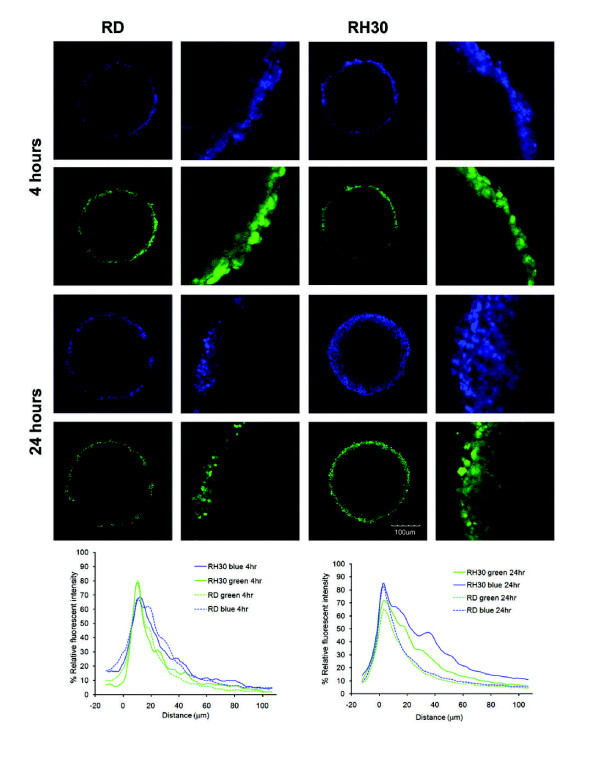
**AS-DACA (1 μM) penetrates further into spheroids made from the RH30 sensitive cell line**. Confocal images of AS-DACA diffusion into spheroids made from the RD and RH30 cell lines after 4 and 24 h. Fluorescence intensity is shown as % Relative Fluorescence Intensity compared to distance penetrated into the spheroid for localization in the nucleus and in cytoplasmic vesicles.

Like the anthracyclines, AS-DACA features a protonation site at the dimethylaminoethyl side chain (Figure 1A) [10]. Fluorescence spectrophotometry indicated that with a reduction in pH, still within a physiological range to 6.2, we see a greater intensity of blue fluorescence from AS-DACA (Figure 4B). Together, the results presented highlight that AS-DACA is sequestered by acidic vesicles of the membrane trafficking system limiting its opportunity to enter the nucleus. Despite this evidence, nuclear accumulation of AS- DACA as evidenced by fluorescent microscopy appeared to be unaltered when examined on a cell-by-cell approach. However, it is thought that the mechanisms have greater relevance in the treatment of solid tumours such as RMS where drug penetration of the tumour mass is obstructed in the outer layers of the tumour [27]. We have shown that impaired penetration of AS-DACA into a solid mass of the resistant RD cells correlates to a reduction of drug accumulation into the nucleus when compared to the sensitive RH30 cells, as evidenced by green fluorescence peaks (Figure 6). From initial drug exposure of the RMS cells, AS-DACA is captured into acidic vesicles where no difference was detectable between cell lines after 10minutes (Figure 4C). It is only after extended exposure to 24 hours that the spheroids indicate a greater penetrance of AS-DACA fluorescence into RH30 spheroids implicating a more efficacious drug-vesicle-transport mechanism in the sensitive cells or a delayed system leading to drug sequestration in the resistant cells.

Knockdown of the major vault protein (MVP) using siRNA disrupted lysosomal uptake of not only doxorubicin, but also the intracellular pH probe LysoSensor and the lysosomal specific antigen LAMP1. This study indicated that MVP was integral in redistributing the drug away from the nucleus to lysosomes [24]. On this basis, a simple explanation of our findings is that the distribution of different vesicle types present in the cell dictates the different response to AS-DACA. Clearly AS-DACA is being captured into acidic cellular compartments (Figure 4) but which are pre-lysosomal, (Figure 5A). Assessment of protein markers associated with vesicles of the membrane trafficking system revealed that reduced expression the early endosomal markers EEA1 and Rab5 was found in the sensitive RH30 cells but increased in the AS-DACA resistant Res-30 to level equal to or above of the RD cell line (Figure 5B). However, the lysosomal markers LAMP1 and Rab5 were unchanged between all cell lines. Whether AS-DACA is merely redistributed away from the nucleus or whether it is effluxed from the cells remains unknown, but unlike MRP1, no significant treatment induced change in expression of these acidic vesicle markers was observed, indicating that this is an intrinsic difference between the RD and Rh30 cell lines.

## Conclusions

The implication from our findings suggests that reduced levels of early endosomal vesicles limit the sequestration and redistribution of AS-DACA away from the nucleus in RH30 cells and hence does not offer any protection from the effects of the drug with topoisomerase poisoning. Taking the evidence as a whole shows that endosomal vesicle trafficking influences the toxicity of AS-DACA in RMS cells and highlights potential new biological targets worthy of further study.

Interestingly, sequestration of AS-DACA was identified through the intracellular visualization of the drug. Most established molecules used to treat cancer however cannot be visualized and hence similar mechanisms may go undetected, only manifesting themselves through a poor tumour response to therapy. The unique fluorescence properties of AS-DACA may be exploited in new and alternative methods of assessing drug penetration, distribution and the overall pharmacokinetics of the drug. Hence, we believe that, regardless of the drugs fate in clinical development, it will provide an invaluable tool to cancer pharmacology researchers interested in understanding the pharmacodynamics and pharmacokinetics of weakly basic cytotoxic compounds.

## Methods

### Drugs

AS-DACA was kindly provided by Professor W.A. Denny from the Auckland Cancer Society Research Centre, University of Auckland, New Zealand. ^®^-Verapamil, mitoxantrone, and doxorubicin were purchased from Sigma-Aldrich Pty Ltd.

### Cell Culture, Cytotoxicity Assays and Development of an AS-DACA- Resistant Cell Line

RD and RH30 rhabdomyosarcoma cell lines were purchased from ATCC, Virginia, USA and were routinely tested for mycoplasma contamination as well as growth consistency prior to establishment and experimentation. The presence of the t[1:13] translocation was confirmed in the Rh30 lines. RD cells were maintained in DMEM medium (Invitrogen) supplemented with 10% fetal bovine serum (FBS) and 2 mM glutamine (Sigma-Aldrich). RH30 cells were maintained in RPMI medium (Invitrogen) and supplemented with 10% FBS and 2 mM glutamine. All cell lines were grown at 37°C in humidified conditions and 5% CO_2_. Three-dimensional multicellular spheroids were cultured by adding 1.5 × 10^5 ^cells/ml to agarose-coated 96-well imaging plates (BD Biosciences), and allowed to aggregate for 96 h, resulting in the formation of a single spheroid per well [28]. MTT viability assays were performed as previously described [8]. Sigmoidal dose-response curves were evaluated using GraphPad Prism 4 by a non-linear regression method, and the concentration of drug that inhibited growth by 50%, the IC_50_, calculated for each agent. An RH30 cell line resistant to AS-DACA was developed by exposing the cells to increasing doses of the drug. Cells, initially treated with AS-DACA at a concentration of 9 nM for 16 h, were exposed to exponentially increasing doses of drug over 6 months, with at least 1 week recovery between treatments. At various stages, MTT viability assays were carried out in order to assess the sensitivity of the cells to AS-DACA. The resulting resistant cell line, named "Res30', was found to tolerate 1 μM AS-DACA, and exhibited this level of resistance throughout the course of all experiments.

### DNA Strand Breaks: Enzyme-Linked ImmunoSorbent Assay [ELISA]

AS-DACA-induced DNA double strand breaks were assessed by measuring the level of phosphorylated histone H2A.X [29] with the anti-phospho-histone γH2A.X (S139) primary monoclonal antibody TK-2F1 using an ELISA kit (Cyclex, Japan) according to the suppliers instructions. Optical absorbance was determined using a Labsystems Multiskan Ascent multi-well plate spectrophotometer at dual wavelengths of 450/540nm. Each cell line was treated for various times with 20 nM AS-DACA, and the level of damage compared, as a percentage, to that resulting from a 48 h exposure of the cells to a saturating dose (4 μM) of AS-DACA.

### Spectrofluorimetry

The influence of pH on the fluorescence properties of AS-DACA was investigated in phosphate buffers, at a concentration of 10 μM, using a Perkin Elmer LS-50B spectrofluorimeter equipped with a Starna Fluorimeter Micro Cell quartz cuvette. Fluorescence emission was measured from 280 to 800 nm with excitation at 260 nm, λmax for AS-DACA absorbance in the far UV.

### Drug Distribution and Intracellular Probes

Cells were passaged at a 1:5 dilution from near-confluent plates onto glass coverslips placed in the bottom of the culture plate. They were subsequently grown for 16 h, and subsequently treated with high dose (1-2 μM) of AS-DACA for short exposures (10 m-4 h), to obtain optimal images with minimal quenching of fluorescence. When required, cells were further incubated for 45 min with LysoTrackerTM Red (Molecular Probes, Invitrogen) at a concentration of 100 nM. Cells were then washed once with ice cold PBS to minimize drug efflux, and immediately mounted onto glass microscope slides using FluorSave mounting media (Calbiochem).

### Indirect Immunofluorescence - MRP1 Expression

Cells grown on glass coverslips were gently washed twice in PBS, fixed permanently by incubating with 4% paraformaldehyde for 20 min, and made permeable by treatment with 0.2% Triton X-100 for 10 min at room temperature. They were then washed 3 times with PBS, and incubated for 1 h at room temperature with MRP1 primary antibody diluted in PBS containing 0.1% BSA. Cells were washed and incubated with Alexa^488^-conjugated donkey anti-mouse (1:500) antibody in 0.1% BSA in PBS for 1 h in the dark. After washing, the cells were counterstained with 10 nM DAPI diluted in PBS, washed, and mounted onto microscope slides.

### Fluorescence Microscopy

The majority of the fluorescent images were obtained using a Jenoptik ProgRes CF Scan digital camera on an Olympus BX50 epifluorescent microscope fitted with a UPlan FL 60 ×/corr 0.65-1.25 oil objective. A U-MWIBA3 filter was used for excitation of AS- DACA within the range 460 to 490 nm, and green emission detected between 510 and 550 nm. Red fluorescence, of doxorubicin and LysoTracker™ Red, was detected above 610 nm using a U-MWIY filter with excitation between 545 and 580 nm. UV excitation of AS-DACA at 330 to 385 nm was achieved using a U-MWV filter with all emissions above 420 nm detected. ProgRes^® ^V2.5 software was used to capture the images. Image enhancement involving colour level adjustment as dictated by the RGB histogram, was carried out in Adobe Photoshop. No other computer enhancement was performed. Extensive photobleaching under continuous UV irradiation proved troublesome for fluorescence imaging of AS-DACA. To address this problem, we used a deconvolution microscope, Delta Vision OMX, which provided precise control of UV excitation exposure and image capture, allowing visualization of the AS-DACA intra-cellular distribution following short exposure times. AS-DACA fluoresced an intense green from excitation at wavelengths between 460 and 490 nm, and blue following excitation between 330 and 385 nm. Images were captured using true colour photography at 600 × magnification, and the displayed pictures are merged images of both filters with no enhancements performed.

### Imaging Drug Penetration into Spheroids

Spheroids were incubated with 10 μM AS-DACA for 24 h and 12-bit confocal images collected on an Olympus FV1000 inverted microscope using an Olympus UPLAPO 10 ×/0.40 air objective lens. Scan rates of 4.0 μs/pixel, 1.5X zoom, Kalman averaging, and sequential collection of the green and blue channels, were applied for all images. Excitation and emission ranges for the 405 nm diode and 488 nm argon lasers, respectively, were as follows: Ex 405 nm, Em 425-475 nm; Ex 488 nm, Em 500-600 nm. Image planes were selected approximately 175 μm inside the spheroid to eliminate edge effects, and reflection imaging was used to ensure 100% laser penetration and emission at that depth within the spheroid. The images were not manipulated in any way, and were analysed using ImageJ software (NIH). A one pixel-wide line was drawn through the centre of the spheroid and the fluorescence intensity was measured. The values were normalised and the average intensity profiles for each wavelength were calculated [28]. Greater than 20 spheroids were measured for each cell line.

### Western Blot Analysis

Western blot analysis was performed using established protocols as previously reported [8]. All antibodies to MRP1, EEA1, Rab5, Rab7, α tubulin [mouse monoclonal] and LAMP1 (rabbit polyclonal) were purchased from Abcam. Primary antibodies were used at the following dilutions: MRP1 1:500, LAMP1 1 μg/ml, EEA1 1:200, Rab5 and Rab7 3 μg/ml, α tubulin 1:3000. Secondary antibodies were donkey anti-rabbit-HRP or donkey anti-mouse-HRP from GE Bioscience diluted to 1:50000.

## Authors' contributions

SJW undertook the majority of the research procedures for this study as part of his postgraduate training. TH contributed the result from the deconvolution microscopy (Figure 4C) whilst NB and TH conducted the experiments exploring spheroid penetration (Figure 6). LPGW is expert in medicinal chemistry and provided valuable direction with interpretation of results with respect to the drug chemistry, in particular the establishment and design of the spectrofluorimetry assessment of AS-DACA (Figure 4B). BWS provided critical comment and review of the study design and outcomes whilst DRC conceived and instigated the entire study and provide overall direction. All authors have read and approved the final manuscript.
